# Bariatric and metabolic surgery improves glycolipid metabolism via bile acid regulation and intestinal barrier repair in T2DM patients

**DOI:** 10.5937/jomb0-58245

**Published:** 2026-01-06

**Authors:** Jingjing Zhang, Shadike Apaer, Shuo Zhang, Guanyou Liang, Tao Li, Xinling Cao

**Affiliations:** 1 State Key Laboratory of Pathogenesis, Prevention and Treatment of High Incidence Diseases in Central Asia, Department of Nephrology, the First Affiliated Hospital of Xinjiang Medical University, Urumqi 830054, China; 2 State Key Laboratory of Pathogenesis, Prevention and Treatment of High Incidence Diseases in Central Asia, Department of Liver Transplantation & Laparoscopic Surgery, the First Affiliated Hospital of Xinjiang Medical University, Urumqi 830054, China

**Keywords:** bariatric and metabolic surgery, type 2 diabetes mellitus, bile acid metabolism, intestinal permeability, TG R5 signalling, GLP-1 secretion, bariatrijska i metabolička hirurgija, dijabetes melitus tipa 2, metabolizam žučnih kiselina, crevna propustljivost, TGR5 signalizacija, sekrecija GLP-1

## Abstract

**Background:**

To observe the changes in bile acid synthase activity, conjugation enzyme gene and intestinal mucosal barrier function (D-LA, Zonulin, MFG-E8) in patients with type 2 diabetes mellitus (T2DM) after bariatric and metabolic surgery (BMS), and to provide an objective opinion on the clinical optimisation of BMS.

**Methods:**

127 patients with T2DM who had received BMS treatment at our hospital from October 2023 to August 2024 were included in the study, and weight loss glucose-lipid metabolism was detected before surgery and 6 months after surgery. Furthermore, the study quantified the expression levels of key enzymes involved in bile acid synthesis and conjugation (CYP7A1, CYP27A1, FXR, FGF19) and markers indicative of intestinal mucosal barrier function (D-LA, Zonulin, MFG-E8).

**Results:**

After BMS, the patient's weight was significantly reduced, and glucolipid metabolism was significantly improved (P&lt; 0.05). In addition, CYP7A1 was decreased, and FXR and FGF19 were elevated in patients after surgery (P &lt; 0.05). Regarding the intestinal mucosal barrier function, D-LA and Zonulin were reduced in patients after surgery (P&lt; 0.05). MFG-E8 was not significantly altered.

**Conclusions:**

BMS can effectively improve glucose-lipid metabolism and reduce body weight in T2DM patients, and its mechanism is related to regulating bile acid metabolism and promoting the recovery of intestinal barrier function.

## Abstract (sr)

**Uvod:**

Cilj studije bio je da se ispita promena aktivnosti enzima za sintezu žučnih kiselina, gena za konjugacione enzime i funkcije crevne mukozne barijere (D-LA, Zonulin, MFG-E8) kod pacijenata sa dijabetesom melitusom tipa 2 (T2DM) nakon bariatrijske i metaboličke hirurgije (BMS), kao i da se pruži objektivno mišljenje o kliničkoj optimizaciji BMS-a.

**Metode:**

U studiju je uključeno 127 pacijenata sa T2DM koji su u periodu od oktobra 2023. do avgusta 2024. godine podvrgnuti BMS tretmanu u našoj ustanovi. Praceni su parametri telesne mase i metabolizma glukoze i lipida pre operacije i šest meseci nakon operacije. Takođe, kvantifikovani su nivoi ekspresije ključnih enzima uključenih u sintezu i konjugaciju žučnih kiselina (CYP7A1, CYP27A1, FXR, FGF19), kao i markeri funkcije crevne mukozne barijere (D-LA, Zonulin, MFG-E8).

**Rezultati:**

Nakon BMS-a, kod pacijenata je došlo do značajnog smanjenja telesne mase i poboljšanja glukolipidnog metabolizma (P&lt; 0,05). Takođe, primeceno je smanjenje ekspresije CYP7A1 i povecanje ekspresije FXR i FGF19 nakon operacije (P&lt; 0,05). Sto se tiče funkcije crevne mukozne barijere, nivoi D-LA i Zonulina su bili značajno sniženi posle operacije (P&lt; 0,05). Nivo MFG-E8 nije se značajno menjao.

**Zaključak:**

Bariatrijska i metabolička hirurgija može da efikasno poboljša glukolipidni metabolizam i smanji telesnu težinu kod pacijenata sa T2DM-om, pri čemu mehanizam delovanja uključuje regulaciju metabolizma žučnih kiselina i podsticanje obnove funkcije crevne barijere.

## Introduction

Diabetes mellitus (DM) represents a spectrum of metabolic disorders unified by the common feature of chronic hyperglycemia [1]. The global prevalence of DM is escalating at an alarming rate, contributing to a substantial burden of metabolic dysfunction and associated complications [2]. Among its subtypes, type 2 DM (T2DM) is the most prevalent and has been identified as a growing public health crisis, particularly in developing countries [3]. Notably, the incidence of T2DM among individuals younger than 40 years has surged two- to threefold in recent decades, highlighting the need for targeted clinical interventions [4]. Studies have shown that abnormal bile acid metabolism is significantly associated with the development of T2DM [5]. Bile acids are not only key mediators of lipid digestion and absorption but also important signalling molecules that regulate glucose-lipid metabolism, insulin sensitivity, and energy homeostasis through farnesol X receptor (FXR) and G protein-coupled bile acid receptor (TGR5) [6] [7]. Among them, abnormal expression of bile acid synthases (e.g., CYP7A1, CYP8B1) and binding enzymes (e.g., bile acid coenzyme A synthase BACS, bile acid acyltransferase BAT) can lead to an imbalance in the composition of the bile acid pool, which can in turn exacerbate insulin resistance by affecting the secretion of intestinal hormones (e.g., GLP-1) and the hepatic gluconeogenesis pathway [8] [9]. However, there is still a gap in the current research on the molecular mechanisms underlying the changes in bile acid synthase and conjugate enzyme activities and their interaction with the intestinal microenvironment in diabetic states.

Meanwhile, impairment of intestinal mucosal barrier function is considered to be an important causative factor of T2DM-related metabolic inflammation [10]. Abnormal expression of intestinal barrier integrity markers such as D-lactic acid (D-LA, an intestinal bacterial metabolite reflecting intestinal permeability), connexin (Zonulin, a switching factor that regulates tight junction proteins), and milk-fat-globule epidermal growth factor 8 (MFG-E8, involved in intestinal epithelial repair and anti-inflammatory regulation) is closely associated with intestinal flora translocation, endotoxemia, and systemic inflammation [11] [12]. Clinical data show that serum D-LA and Zonulin levels are significantly elevated and MFG-E8 expression is down-regulated in patients with T2DM, suggesting that intestinal barrier disruption may exacerbate glucose metabolism disorders through the »gut-hepatic axis« and »gut-islet axis« [13]. However, the dynamics of these biomarkers in diabetes progression and their synergistic effects with bile acid metabolism have not been fully elucidated.

Central to the management of T2DM is the optimisation of glycemic control. Bariatric and metabolic surgery (BMS), renowned for its durable and profound glucose-lowering effects, has become a cornerstone therapy for T2DM patients with a body mass index (BMI) exceeding 30 kg/m^2^ [14]. Despite evidence linking bile acid dysregulation to T2DM, the molecular mechanisms of BMS on bile acid synthase activity and its interaction with intestinal barrier repair remain poorly understood. Moreover, the differential effects of SG and RYGB on these pathways are yet to be systematically explored. We hypothesised that BMS improves glucose and lipid metabolism by regulating bile acid synthesis (via CYP7A1 suppression and FXR/FGF19 activation) and restoring intestinal barrier integrity (via D-LA/Zonulin reduction and MFG-E8 upregulation).

Based on this, this study aimed to investigate the effects of BMS on bile acid synthase activity, binding enzyme gene expression, and intestinal mucosal barrier function markers (D-LA, Zonulin, MFG-E8) in patients with T2DM and to analyse its association with the improvement of glucose metabolism. The results of the study are expected to provide new evidence to reveal the molecular mechanism of BMS in the treatment of diabetes and to lay a theoretical foundation for the development of novel therapeutic strategies targeting bile acid metabolism and intestinal barrier repair.

## Materials and methods

### Study design

This investigation was designed as a prospective analysis. Following power analysis, 127 individuals diagnosed with T2DM who underwent BMS (sleeve gastrectomy (SG) or Roux-en-Y gastric bypass (RYGB)) at our hospital from October 2023 to August 2024 were recruited. The sample size was determined based on previous studies [15], assuming an effect size of 0.6 (Cohen's d) for bile acid-related biomarkers, with α = 0.05 and β = 0.2. A minimum of 55 patients per group was required; we enrolled 68 (SG) and 59 (RYGB) to account for potential attrition. All enrolled patients were monitored for a minimum duration of 6 months following surgery. Follow-up evaluations were conducted through scheduled monthly hospital visits to ensure comprehensive postoperative assessment. The information of the patients is shown in Table 1. Ethical approval for this study was duly obtained from the Ethics Committee of our institution, and all participants provided their informed consent prior to study enrollment.

**Table 1:** Baseline information.

Groups	Age	Sex		Duration<br>of T2DM<br>(years)	Complications		
		Male	Female		Fatty liver	High blood<br>pressure	None
SG group<br>(68)	50.16±4.78	32 (47.06%)	36 (52.94%)	8.69±1.47	16 (23.53%)	24 (35.29%)	28 (41.18%)
RYGB group<br>(59)	51.17±6.72	26 (52.94%)	33 (55.93%)	8.63±1.87	12 (20.34%)	24 (40.68%)	23 (38.98%)
95%CI	-1.022 to<br>3.038	-		-0.6518 to<br>0.5237	-		
*t* (χ^2^)	0.9836	0.1139		0.2157	0.4260		
*P*	0.3277	0.7357		0.8296	0.8082		

### Inclusion and exclusion criteria

Inclusion criteria: Eligible participants had a confirmed diagnosis of T2DM, a BMI 230 kg/m^2^, and were scheduled for their first BMS (done by the same surgical team).

Exclusion criteria: Individuals were excluded if they presented with gastrointestinal disorders, malignancies, impaired organ function, a history of gastric surgeries, severe gastroesophageal reflux, secondary obesity, a history of alcohol or drug abuse, or psychiatric or cognitive impairments.

### Laboratory investigations

A series of laboratory tests were conducted to assess the glucose-lipid metabolism profiles of patients at two key time points: baseline (upon admission) and 6 months postoperatively. Key parameters included fasting blood glucose (FBG), glycated haemoglobin (HbA1c), homeostatic model assessment of insulin resistance (HOMA-IR), total cholesterol (TC), triglycerides (TG), low-density lipoprotein cholesterol (LDL-C), and high-density lipoprotein cholesterol (HDL-C). These analyses were carried out using a Mindray fully automated biochemical analyser. Additionally, waist circumference, body weight, and BMI were recorded.

Serum concentrations of D-lactic acid (D-LA), Zonulin, and milk fat globule-EGF factor 8 (MFG-E8) were quantified using enzyme-linked immunosorbent assay (ELISA). ELISA kits for D-LA (LK-D001, Hangzhou Lianke), Zonulin (LK-Z002), and MFG-E8 (LK-M003) were used.

The expression levels of genes involved in bile acid synthesis and conjugation, namely cytochrome P450 family 7 subfamily A member 1 (CYP7A1), cytochrome P450 family 27 subfamily A member 1 (CYP27A1), Farnesoid X receptor (FXR), and fibroblast growth factor 19 (FGF19), were determined via polymerase chain reaction (PCR). ExoQuick extracted exosomes, and total RNA was extracted using the TRIzol-LS method. cDNA was synthesised by reverse transcription: Prime Script RT kit. Primer sequences were designed and constructed by Hunan FengHui Biotechnology Co Ltd (Table 2, Catalogue No. FH-PR-2024). Primer specificity was confirmed via melting curve analysis (single peak) and Sanger sequencing. PCR efficiency (90-110%) was validated using standard curves. Amplification conditions: pre-denaturation at 95°C for 5 min, 95°C for 10 s, 60°C for 30 s, 40 cycles (SYBR Green method), standardised by the internal reference GAPDH, and relative expression was analysed by 2^-ΔΔCt^ method.

**Table 2:** Sequence of primers.

	F (5'-3')	R (5'-3')
CYP7A1	CAGCAACTAAACAACCTGCCG	TGGTAGAGCTCACAGGACCA
CYP27A1	CTGCCTGGAGAAAGTGCTTC	GGAACACCAGGATGCTGAAG
FXR	CAGCCTCATCCACATCCTCT	TCCACGATGTTCTCCTCCAC
FGF19	CAGCCAGGT GTCCTACATCA	TGGCAGAGACACGGTAGATG
GAPDH	GGAGCGAGATCCCTCCAAAAT	GGCTGTTGTCATACTTCTCATGG

### Statistical methods

Data analyses were performed using SPSS software (version 25.0). Categorical variables, such as gender distribution, were compared using the chi-square test. For continuous variables, including age and bile acid-related metrics, independent samples t-tests were utilised for intergroup comparisons, while paired t-tests were applied for intragroup comparisons before and after surgery. Bonferroni correction was applied for multiple comparisons (adjusted α = 0.05/8=0.00625). A significance level of *P*<0.05 was set to denote statistically significant differences.

## Results

### Changes in glucose-lipid metabolism profiles

**Figure 1:**
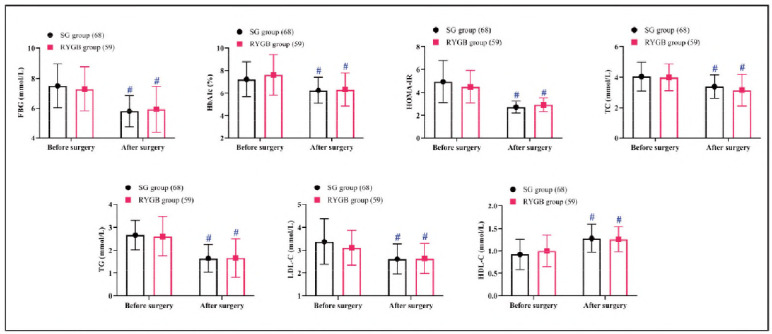
Comparison of glycolipid metabolism.<br>Note: #P < 0.05 compared to before surgery

Preoperative and postoperative assessments of FBG, HbA1c, HOMA-IR, TC, TG, LDL-C, and HDL-C showed no significant differences between the groups (*P*>0.05). Postoperatively, both groups demonstrated significant reductions in all measured parameters except HDL-C, which increased (*P*<0.05) (Figure 1).

### Changes in weight loss

**Figure 2:**
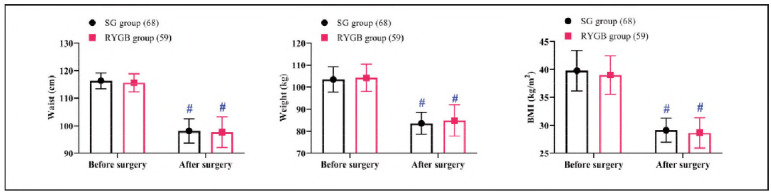
Comparison of weight loss.<br>Note: #P < 0.05 compared to before surgery

Baseline and postoperative measurements of waist circumference, body weight, and BMI were similar between the groups (*P*>0.05). Both groups achieved significant reductions in these parameters postoperatively (*P*<0.05, Figure 2).

### Changes in bile acid levels

**Figure 3:**
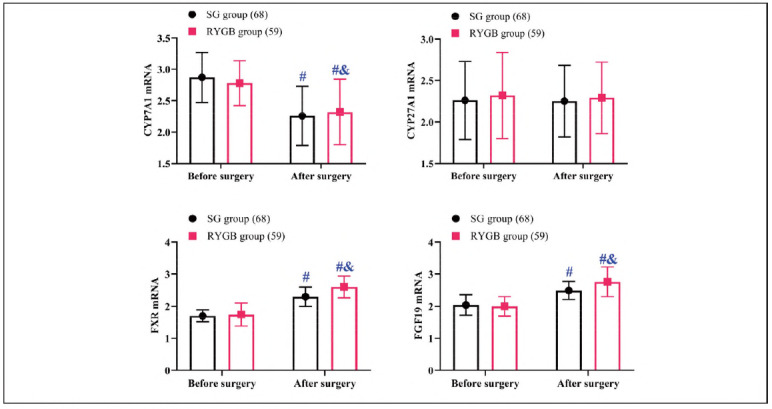
Comparison of bile acid metabolism.<br>Note: #P < 0.05 compared to before surgery, & P < 0.05 compared to SG group

Evaluation of bile acid-related biomarkers indicated no significant differences in the preoperative expression of genes involved in bile acid synthesis and conjugation between the two cohorts (*P*>0.05). Following surgery, CYP27A1 levels remained stable in both groups compared to preoperative values (*P*>0.05). In contrast, CYP7A1 expression decreased significantly, with the RYGB group demonstrating a more marked decline than the SG group (*P*<0.05). Conversely, FXR and FGF19 levels exhibited a postoperative increase, with the RYGB group achieving higher levels than the SG group (*P*<0.05) (Figure 3).

### Changes in intestinal mucosal barrier function

**Figure 4:**
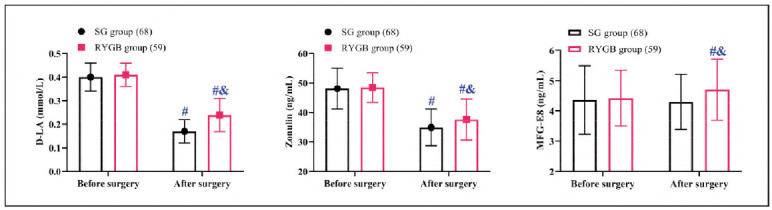
Comparison of intestinal mucosal barrier function.<br>Note: #P < 0.05 compared to before surgery, & P < 0.05 compared to SG group

In terms of intestinal mucosal barrier function, baseline measurements of D-LA, Zonulin, and MFG-E8 were comparable between the two groups (*P*>0.05). Postoperatively, MFG-E8 levels in the SG group showed no significant change (*P*>0.05), whereas in the RYGB group, a considerable upsurge was observed when compared to the baseline levels (*P*<0.05). Both groups experienced reductions in D-LA and Zonulin levels after surgery, with the SG group achieving lower levels than the RYGB group (*P*<0.05) (Figure 4).

## Discussion

Focusing on the effects of BMS on key enzymes of bile acid metabolism (CYP27A1), bile acid signalling pathway (FXR/FGF19 axis), and intestinal mucosal barrier function (D-LA, Zonulin, and MFG-E8), this study reveals the roles of these targets in the pathology of T2DM and the potential mechanism of surgical interventions, which provides a new theoretical basis for optimising BMS.

First, we observed that both body weight and glucolipid metabolism were effectively improved in patients after BMS, which triggers a series of metabolic adaptive responses by directly altering the anatomy of the gastrointestinal tract [16]. Insulin sensitivity was significantly improved in postoperative patients, as evidenced by a decrease in fasting glucose, HbA1c, and HOMA-IR, which was accompanied by an improvement in the lipid profile (e.g., lower triglycerides and higher HDL-C). This effect was not only associated with weight loss but also relied on the synergistic effect of reprogramming bile acid metabolism and repair of intestinal barrier function.

Thus, a more in-depth analysis was carried out for bile acids and intestinal barrier function. First, it was seen that the total bile acid pool and the proportion of secondary bile acids increased significantly after BMS, especially in the RYGB group. The expression of the classical synthetic pathway rate-limiting enzyme CYP7A1 was suppressed. In contrast, the expression of the alternative pathway enzyme CYP27A1 was upregulated, suggesting that the surgery may optimise the composition of bile acids by altering their synthetic pathways (e.g., increasing the proportion of deoxycholic acid (DCA). The activation of CYP27A1 further promotes bile acid binding to the intestinal FXR receptor, driving the secretion of FGF19 [17]. FGF19 acts on the liver through the portal circulation to inhibit key enzymes of gluconeogenesis (e.g., PEPCK) and promote hepatic glycogen synthesis, as well as to improve lipid metabolism by inhibiting fatty acid synthase (FAS) [18] [19]. In addition, the activation of FXR can also upregulate the expression of intestinal epithelial tight junction proteins (e.g., ZO-1, Claudin-4), which can indirectly repair the intestinal barrier function and form a positive feedback regulation of the »bile acid-intestinalhepatic axis«[20].

On the other hand, it has been clinically confirmed that patients with T2DM commonly suffer from intestinal mucosal barrier damage, as evidenced by elevated serum D-LA and Zonulin levels and down-regulation of MFG-E8 expression [21]. The elevation of D-LA, as a marker of intestinal permeability, reflects bacterial metabolites translocation to the circulatory system, which triggers endotoxemia and systemic inflammation (e.g., elevated levels of IL-6 and TNF-α), thereby exacerbating insulin resistance [22] [23]. levels are elevated, which in turn exacerbates insulin resistance [23]. In this study, the significant decrease in D-LA and Zonulin after BMS suggests the restoration of intestinal barrier integrity, which may be related to the following mechanisms:(1) Bile acid-TGR5 signalling: secondary bile acids (e.g., lithocholic acid (LCA)) enhance tight junction protein assembly by activating intestinal epithelial TGR5 receptor and inhibit the NF-B-mediated inflammatory pathway [24]. (2) Up-regulation of MFG-E8: MFG-E8, as an intestinal repair factor, promotes epithelial cell migration and mucus layer regeneration through integrin signalling while inhibiting macrophage M1 polarisation and reducing local inflammation in the intestine [25]. (3) The restoration of intestinal barrier function reduces endotoxin entry into the bloodstream and alleviates metabolic inflammation, which in turn improves insulin signalling in adipose tissue [26].

In the present study, differences in the effects of the two BMS procedures on T2DM were seen, and the reasons for this may be analysed to be related to the different targets of action of the two procedures. RYGB directly enhances bile acid-FXR/FGF19 axis activity through intestinal anatomical remodelling (e.g., biliopancreatic shunt, rapid entry of food into the distal ileum) [27]. Exposure of terminal ileal L cells to high concentrations of bile acids promotes GLP-1 secretion, which synergistically enhances insulin secretion and sensitivity via the central nervous system (e.g., hypothalamus) and peripheral tissues (e.g., pancreatic β-cells) [28]. In addition, RYGB upregulates explicitly the expression of MFG-E8, which may be associated with its promotion of intestinal stem cell proliferation and anti-apoptotic effects [29]. RYGB's anatomical remodelling (e.g., ileal bile acid exposure) directly activates FXR, enhancing MFG-E8 expression through integrin signalling. In contrast, SG primarily reduces systemic inflammation, indirectly stabilising the intestinal barrier without direct FXR activation. SG is dominated by reduced gastric volume and inhibition of fundic Ghrelin secretion [30]. Decreased Ghrelin levels attenuate hepatic lipid accumulation and oxidative stress while indirectly improving metabolism by modulating the hypothalamic ingestive centre via vagal signalling [31]. Although MFG-E8 remained unchanged in the SG group, the reduction in systemic inflammation (e.g., IL-6, TNF-α) may indirectly stabilise intestinal barrier function via macrophage M2 polarisation. Notably, neither RYGB nor SG exert a conspicuous influence on CYP27A1. This suggests that the regulation of CYP27A1 might be contingent upon non-canonical pathways, such as those associated with vitamin D metabolism or post-transcriptional modifications mediated by inflammatory mediators [32]. Evidently, further research is warranted to validate this hypothesis.

However, this study has the following limitations: the specific effects of different bile acid monomers (e.g., ursodeoxycholic acid (UDCA), DCA) on the signalling pathway were not differentiated; the interactive effects of changes in the intestinal flora and bile acid metabolism need to be further resolved. Future studies should determine the impact of individual bile acids (e.g., DCA, LCA using metabolomic profiling) and the lack of long-term follow-up data to assess the durability of metabolic improvement. Future studies should extend the follow-up to 1-2 years to assess durability and integrate metagenomic sequencing to explore the gut microbiota-bile acid axis.

## Conclusion

BMS reverses glucose-lipid metabolism in T2DM patients by activating the FXR/FGF19 signalling pathway and repairing the intestinal mucosal barrier function (D-LA, Zonulin, MFG-E8). RYGB is based on the »bile acid-gut-liver axis« as the core mechanism, while SG is more dependent on the reduction of systemic metabolic load. In the future, we need to combine multi-omics techniques to analyse the specific regulation of the metabolic microenvironment by different surgical procedures in order to promote the optimisation of individualised metabolic surgical strategies.

## Dodatak

### Availability of data and materials

The data that support the findings of this study are available from the corresponding author upon reasonable request.

### Funding

This study was supported by the State Key Laboratory of Pathogenesis, Prevention and Treatment of High Incidence Diseases in Central Asia, Xinjiang Medical University (No. SKL-HIDCA-2023-26); Xinjiang Medical University Student Innovation and Entrepreneurship Training Program (No. S202410760073).

### Acknowledgements

Not applicable.

### Conflict of interest statement

All the authors declare that they have no conflict of interest in this work.
